# Draft Genome Sequence of a Campylobacter coli WL22 Isolate Possessing *erm*(B) with *gyrA* Mutations, Isolated from Poultry in China

**DOI:** 10.1128/MRA.00183-21

**Published:** 2021-05-06

**Authors:** Lin Wu, Xia Zhai, Yaojian Jin, Hongqiang Lou, Junwei Huang

**Affiliations:** a Department of Medical College, Jinhua Polytechnic, Jinhua, Zhejiang, China; b Department of Clinical Laboratory, Affiliated Jinhua Hospital, Zhejiang University School of Medicine, Jinhua, Zhejiang, China; Loyola University Chicago

## Abstract

Campylobacter coli is a major foodborne pathogen worldwide that causes campylobacteriosis cases in humans and is an emerging threat in developing countries. The rapid dissemination of the macrolide resistance gene *erm*(B) poses a significant threat to the clinical therapy of campylobacteriosis. Here, we report the draft genome sequences of one Campylobacter coli strain possessing *erm*(B), isolated from the cecal contents of poultry in Jinhua, China.

## Abstract (precis)

Campylobacter coli is a major foodborne pathogen worldwide that causes campylobacteriosis cases in humans and is an emerging threat in developing countries. The rapid dissemination of the macrolide resistance gene *erm*(B) poses a significant threat to the clinical therapy of campylobacteriosis. Here, we report the draft genome sequences of one Campylobacter coli strain possessing *erm*(B), isolated from the cecal contents of poultry in Jinhua, China.

Campylobacter coli is a common foodborne pathogenic bacterium that causes bacterial gastroenteritis in humans (1). Poultry are believed to be the most important sources of C. coli human infections. The rapid dissemination of the macrolide resistance gene *erm*(B) will likely compromise the efficacy of macrolides as the treatment of choice for campylobacteriosis (2).We describe here the draft genome sequences of one Campylobacter coli WL22 strain (GONG3) possessing *erm*(B) with *gyrA* mutations.

## ANNOUNCEMENT

Campylobacter coli is a common foodborne pathogenic bacterium that causes bacterial gastroenteritis in humans (1). Poultry are believed to be the most important source of C. coli human infections. The rapid dissemination of the macrolide resistance gene *erm*(B) will likely compromise the efficacy of macrolides as the treatment of choice for campylobacteriosis (2). We describe here the draft genome sequences of one Campylobacter coli WL22 strain (GONG3) possessing *erm*(B) with *gyrA* mutations.

*Campylobacter* sp. isolation from the cecal contents of poultry in slaughterhouses was performed using the *Campylobacter* isolation kit incorporating a membrane filter method (ZC-CAMPY-002; Qingdao Sinova Biotechnology Co., Ltd., Qingdao, China), according to the manufacturer’s instructions. Species identification of C. coli strain GONG3 was conducted with the matrix-assisted laser desorption ionization–time of flight mass spectrometry (MALDI-TOF MS) system at the Department of Medical College of Jinhua Polytechnic in Jinhua, China in 2019. To isolate the genome DNA, the strains were preenriched for 24 h at 42°C in Bolton broth containing *Campylobacter* growth supplements, under microaerobic conditions (5% O_2_, 10% CO_2_, and 85% N_2_).One-hundred-microliter drops of the preenrichment were plated onto the surface of a Columbia blood agar plate. These plates were further cultured overnight at 42°C under microaerobic conditions.

Genomic DNA of strain GONG3 was extracted using a DNeasy blood and tissue kit (Qiagen, Germany), according to the manufacturer’s instructions. Genomic DNA purity and concentration were evaluated by NanoDrop spectrophotometer and measured by a fluorometer (Qubit; ThermoFisher). The DNA library was prepared using a Nextera XT DNA library preparation kit (Illumina, Inc., Cambridge, UK), and genomic DNA was sequenced on an Illumina NovaSeq instrument with a 150-bp paired-end approach at a depth of approximately 200×, which yielded 3,287,640 paired-end raw reads. The quality of sequencing and trimming were verified with FastQC v0.11.7, while low-quality sequences and Illumina PCR adapter sequences were removed with Trimmomatic v0.36 (3). All good-quality paired reads of C. coli GONG3 were assembled using the SPAdes genome assembler v3.12.0 with default settings (4), which yielded 38 contigs with an *N*_50_ value of 269,170 bp, and the total number of assembled bases was 1,680,364 bp. The overall GC content of the C. coli GONG3 strain was 31.44%. Rapid Annotations using Subsystems Technology (RAST) showed that 1,764 coding sequences (CDS) and 42 RNAs were observed in the genome (5).

Whole-genome sequence data analyses were performed using bioinformatics tools (MLST v2.0 and PathogenFinder v1.1) with default parameters available from the Center for Genomic Epidemiology (6). MLST v2.0 demonstrated that C. coli GONG3 belongs to multilocus sequence typing (MLST) sequence type 872 (ST872). The PathogenFinder analysis revealed a pathogenic potential for C. coli GONG3 as a human pathogen. This strain matched 66 pathogenic families (90.8%) indicating a high risk for human infections. C. coli GONG3 carried cytolethal distending toxin (CDT) composed of *cdtA*, *cdtB*, and *cdtC* genes.

Resistance genes in strain GONG3 were screened using RGI 5.1.1 (7), which showed that it contained the resistance genes to a macrolide [*erm*(B)], beta-lactam (*bla*_OXA-451_), aminoglycosides [*aac(6')-aph(2')*, *ant(6)-Ia*, *aph(2')-If*, and *aph(3')-III*], and tetracycline (*tetO*) and the fluoroquinolone resistance-related Thr-86-Ile substitution in *gyrA*. It is alarming that *erm*(B) is always associated with multidrug resistance genomic islands (MDRGIs), which confer resistance to multiple classes of antibiotics, including aminoglycosides, fosfomycin, and tetracyclines (8).

The presented genome sequences of *erm*(B)-positive Campylobacter coli strain WL22 could provide valuable knowledge for understanding the macrolide resistance and genetic characteristics of C. coli.

### Data availability.

The complete genome sequences of the C. coli WL22 isolate reported here have been deposited at DDBJ/ENA/GenBank under the accession no. JACZZI000000000. The raw data have been deposited at the SRA under the accession no. SRR12762486.
